# Identification of key ferroptosis-related genes and therapeutic target in nasopharyngeal carcinoma

**DOI:** 10.3389/fgene.2025.1595456

**Published:** 2025-07-31

**Authors:** Yuanyuan Gu, Yaozhuang Zhou, Chunhua Xie, Guangyao He, Maosheng Zhang

**Affiliations:** ^1^ School of Information and Management, Guangxi Medical University, Nanning, China; ^2^ Key Laboratory of Early Prevention and Treatment for Regional High Frequency Tumor (Guangxi Medical University), Ministry of Education, Nanning, Guangxi, China; ^3^ State Key Laboratory of Targeting Oncology, Guangxi Medical University, Nanning, Guangxi, China

**Keywords:** nasopharyngeal carcinoma, ferroptosis, immune infiltration, drug prediction, enrichment analysis

## Abstract

**Objective:**

Nasopharyngeal carcinoma (NPC) is a malignant tumor, but the role of ferroptosis-related genes in NPC remains unclear. This study aimed to identify ferroptosis-related therapeutic targets and explore their mechanisms in NPC.

**Method:**

NPC datasets and ferroptosis-related genes were obtained from GEO and FerrDB, respectively. Ferroptosis-related differentially expressed genes (DEGs) were identified, and Weighted Gene Co-expression Network Analysis (WGCNA) was used to pinpoint disease-related genes. Four machine learning algorithms screened hub genes, validated by ROC curves. Functional enrichment (GSEA, GSVA), drug prediction (DGIdb), immune infiltration analysis (CIBERSORT), and single-cell RNA sequencing (scRNA-seq) were performed.

**Result:**

From 3405 DEGs, 90 ferroptosis-related genes were identified, enriched in ferroptosis, IL-17, and p53 signaling pathways. WGCNA revealed 34 disease-related genes, and four hub genes (TBK1, KIF20A, SLC16A1, QSOX1) were selected, showing high diagnostic efficacy. GSEA/GSVA highlighted pathway differences between high/low expression groups. Eleven potential drugs were predicted, and immune analysis indicated increased macrophage M1 and neutrophil infiltration. scRNA-seq validated hub gene expression profiles.

**Conclusion:**

This study identified four ferroptosis hub genes in NPC, offering insights into its molecular mechanisms and potential diagnostic/therapeutic targets.

## Introduction

NPC is a malignant tumor originating from the epithelial cells of the nasopharynx, prone to metastasis, and is most commonly found in southern China (Chua et al., 2016; Su et al., 2022). It is estimated that in 2022, there were 120,041 new cases and 73,476 deaths globally due to NPC (Freddie et al., 2024). The primary treatment for NPC is radiotherapy or chemoradiotherapy (Bossi et al., 2021). Although the survival rate for chemoradiotherapy is satisfactory (85%–90% over 5 years) (Ma et al., 2012; Wirth et al., 2010), 8%–10% of patients experience recurrence and tumor metastasis (Vermorken et al., 2013; Xue et al., 2013). Therefore, there is an urgent need to further explore targeted biomarkers for NPC to assist in the development of new therapeutic strategies for the disease.

Ferroptosis is a novel form of cell death caused by oxidative damage, which was first proposed in 2012. It is primarily mediated by iron ion-induced oxidative damage, lipid peroxidation, and cell membrane damage (Stockwell et al., 2017). Through the distinctive features of ferroptosis, iron ions can be utilized to promote ferroptosis in cancer cells, enabling precise cancer treatment (Hassannia et al., 2019). Ferroptosis plays a significant role in various diseases, including cardiovascular diseases, kidney diseases, and malignant tumors, and has become an important area of focus in cancer research (Lei et al., 2022; Liu et al., 2023). Ferroptosis also plays a critical role in NPC. Chen et al. identified that ACSL4 inhibits the pathogenesis of NPC through ferroptosis and crosstalk with macrophages by detecting ACSL4 expression in NPC cell lines and xenograft mice, providing a potential direction for NPC therapy (Chen et al., 2023). Zhou et al. demonstrated that P4HA1 activates HMGCS1 to promote resistance to ferroptosis and progression in nasopharyngeal carcinoma (Zhou et al., 2023). Xu et al. (2021) demonstrated that itraconazole could reduce the activity of NPC stem cells by increasing the concentration of intracellular iron and lipid peroxides in lysosomes. Lactotransferrin (LTF) has been reported to be highly expressed in NPC cells, and its overexpression inhibits NPC cell proliferation by regulating the MAPK/AKT pathway, which serves as a crucial pathway for tumor radiosensitization (Zhou et al., 2008; Zhang et al., 2011; Deng et al., 2013; Song et al., 2019). NRF2 is recognized as a master regulator of antioxidant responses during ferroptosis, as numerous downstream target genes under its control are responsible for preventing redox imbalance in cancer cells (Dodson et al., 2019). Zhang et al. (2017) reported that reducing NRF2 levels and promoting ROS generation sensitized NPC cells to radiotherapy (RT). In summary, ferroptosis plays a crucial role in the development of NPC.

Although there are few studies have focused on ferroptosis-related genes in NPC, research on the mechanisms of ferroptosis treatment targets in NPC is still relatively scarce. Therefore, this study aims to analyze existing datasets related to NPC to identify hub genes associated with ferroptosis and NPC, and further investigate the relationships between these hub genes, immune cells, and pathways. This research seeks to provide new insights for both clinical and basic research on NPC, potentially aiding clinicians in developing personalized treatment plans for NPC patients.

## Result

### Identification of differentially expressed ferroptosis-related genes in NPC

**FIGURE 1:**
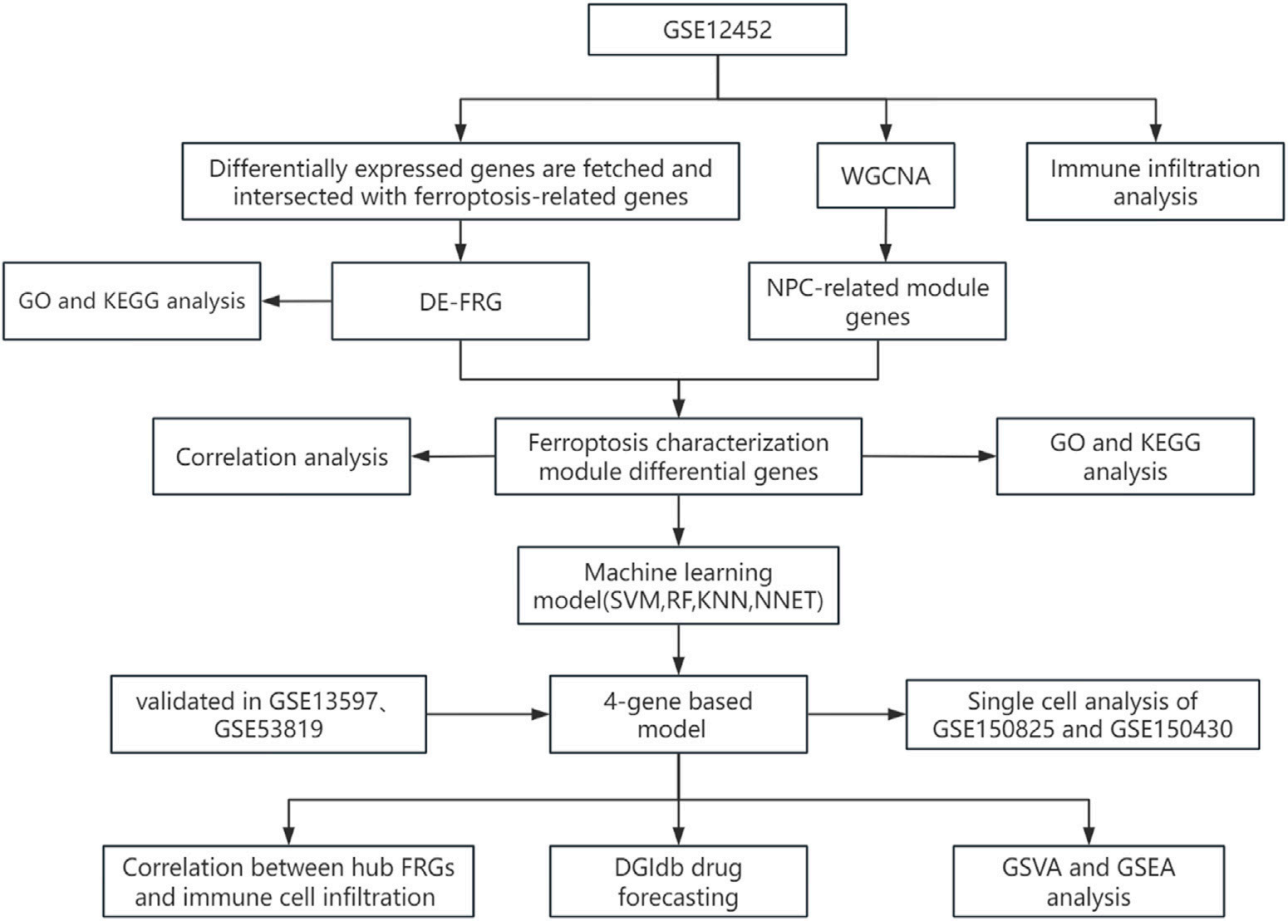
The workflow chart of the whole analysis process in this study.

**FIGURE 2:**
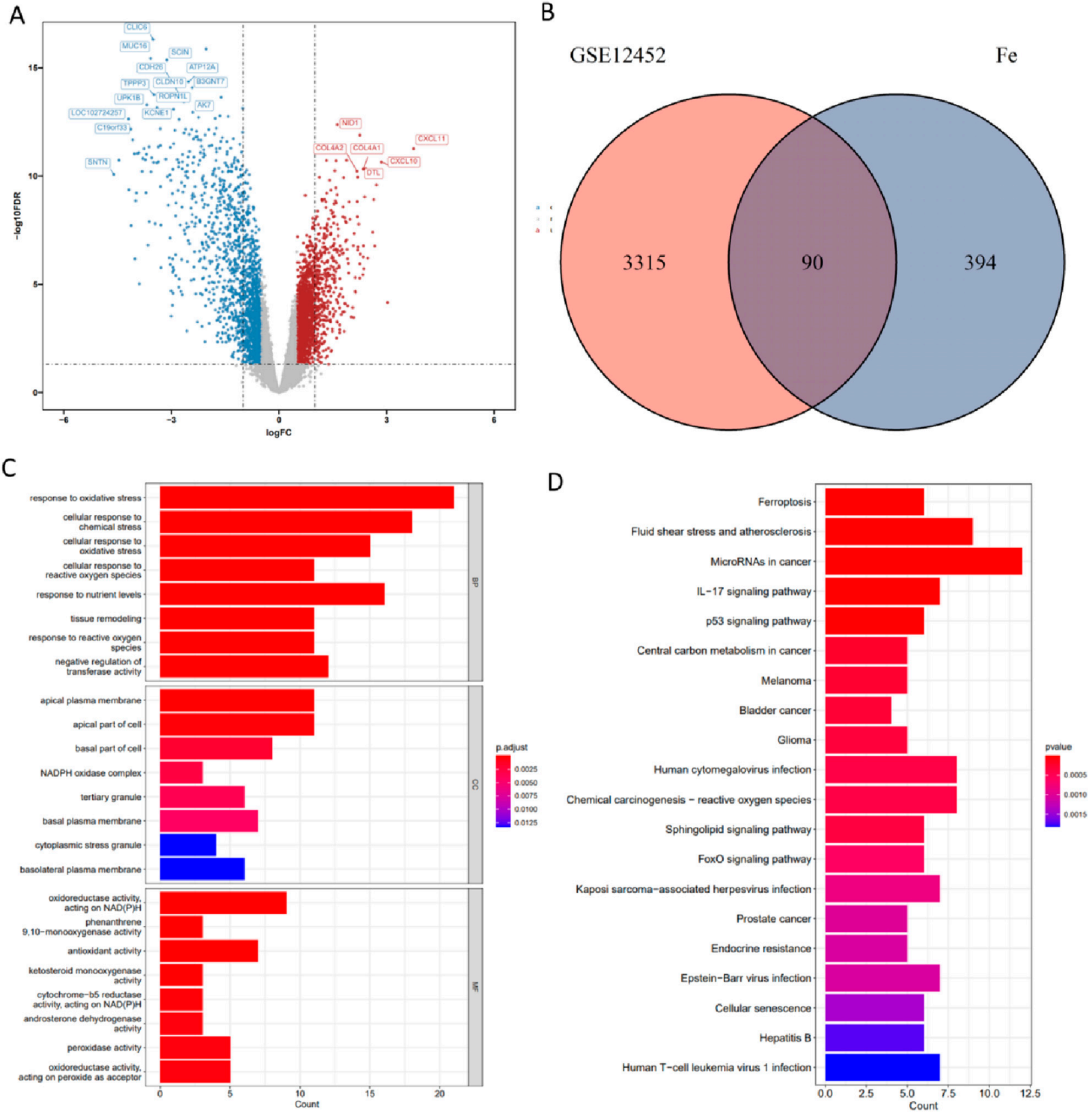
Analysis of DE-FRGs in NPC. **(A)** Volcano plot showing differentially expressed genes in GSE12452. **(B)** Venn diagram of DE-FRGs. **(C)** The bar chart of GO enrichment results. **(D)** The bar plot of KEGG pathway analysis.

In this study, we systematically investigated the association between ferroptosis-related genes and nasopharyngeal carcinoma (Figure 1). A total of 3,405 DEGs were screened from the GSE12452 dataset, with 1,808 genes upregulated and 1,597 genes downregulated in NPC (Figure 2A). Ferroptosis-related genes were downloaded from the FerrDb database, including 369 driver genes, 348 suppressor genes, and 11 marker genes. After removing duplicate annotations, 484 ferroptosis-related genes were obtained. The intersection of these 484 ferroptosis-related genes with the 3,405 DEGs yielded 90 differentially expressed ferroptosis-related genes (DE-FRGs), including 53 upregulated and 37 downregulated genes (Figure 2B).

### Enrich analysis on differentially expressed ferroptosis-related genes

Gene Ontology (GO) and Kyoto Encyclopedia of Genes and Genomes (KEGG) enrichment analyses were performed on the differentially expressed ferroptosis-related genes. The GO biological process (GO-BP) analysis revealed that the genes were primarily enriched in response to oxidative stress, cellular response to chemical stress, cellular response to reactive oxygen species (ROS), cellular response to oxidative stress and response to nutrient levels. The GO cellular component (GO-CC) analysis indicated that these genes were mainly associated with the apical plasma membrane, apical part of cell and basal part of cell. The GO molecular function (GO-MF) analysis showed that the genes were enriched in oxidoreductase activity, NAD(P)H, antioxidant activity, peroxidase activity, and oxidoreductase activity, acting on peroxide as accepter (Figure 2C). Additionally, KEGG pathway analysis demonstrated that these differentially expressed ferroptosis-related genes (DE-FRGs) were enriched in pathways related to microRNAs in cancer, fluid shear stress and atherosclerosis, IL-17 signaling pathway, p53 signaling pathway, and ferroptosis (Figure 2D).

### Construction of co-expression network, module feature selection, and identification of differential genes associated with ferroptosis

**FIGURE 3:**
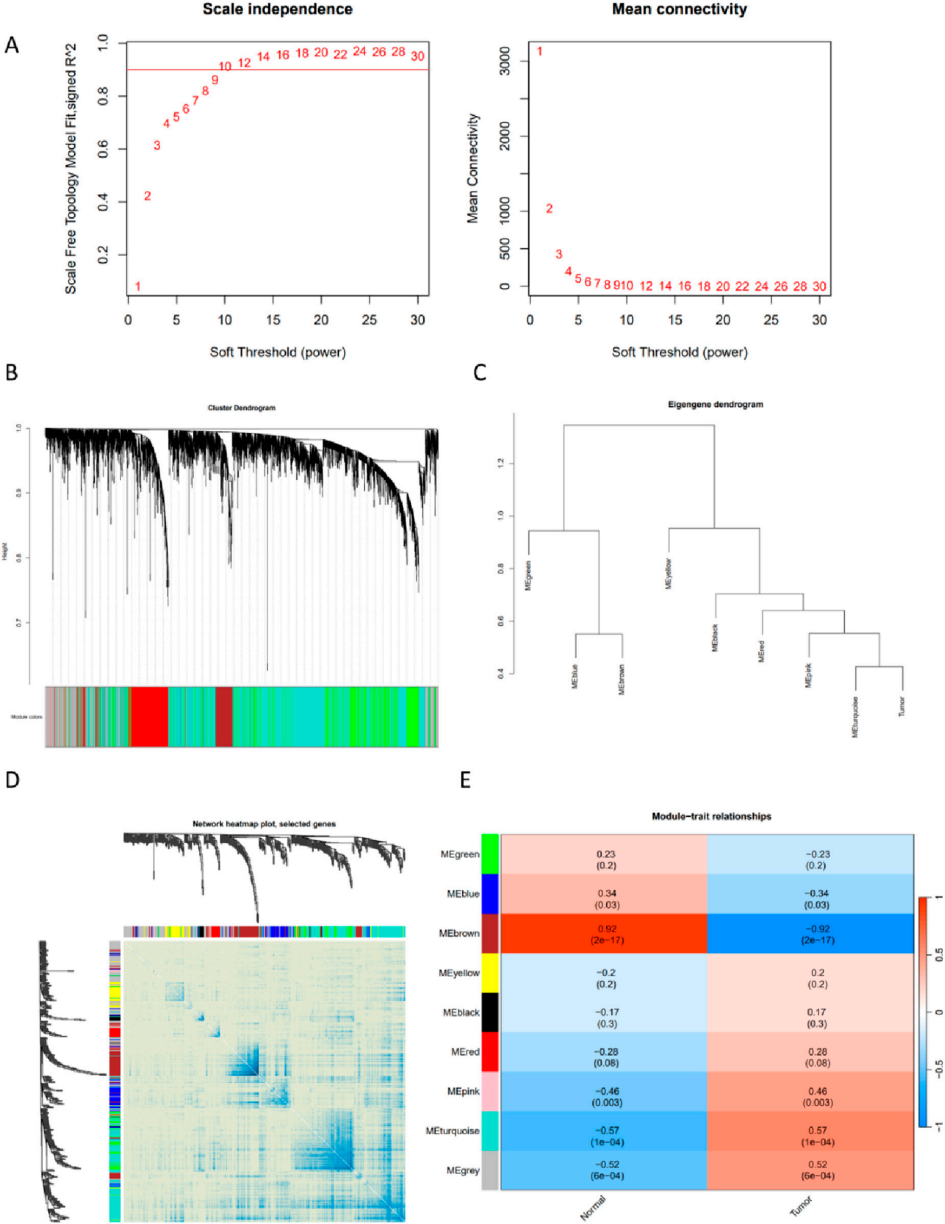
WGCNA identified the characteristic module genes in GSE12452. **(A)** Network topology analysis for various soft-threshold powers. The left panel shows the optimal soft-threshold power selected based on the scale-free topology index. The right panel displays the average connectivity analysis for different soft-threshold powers. **(B)** Clustering dendrogram of co-expression modules based on topological overlap, with different colors representing distinct co-expression modules determined by dynamic tree cutting. **(C)** Representative clusters of module feature genes and microarray sample traits. **(D)** Heatmap visualization of the co-expression network. **(E)** Correlation heatmap between module feature genes and clinical phenotypes.

**FIGURE 4:**
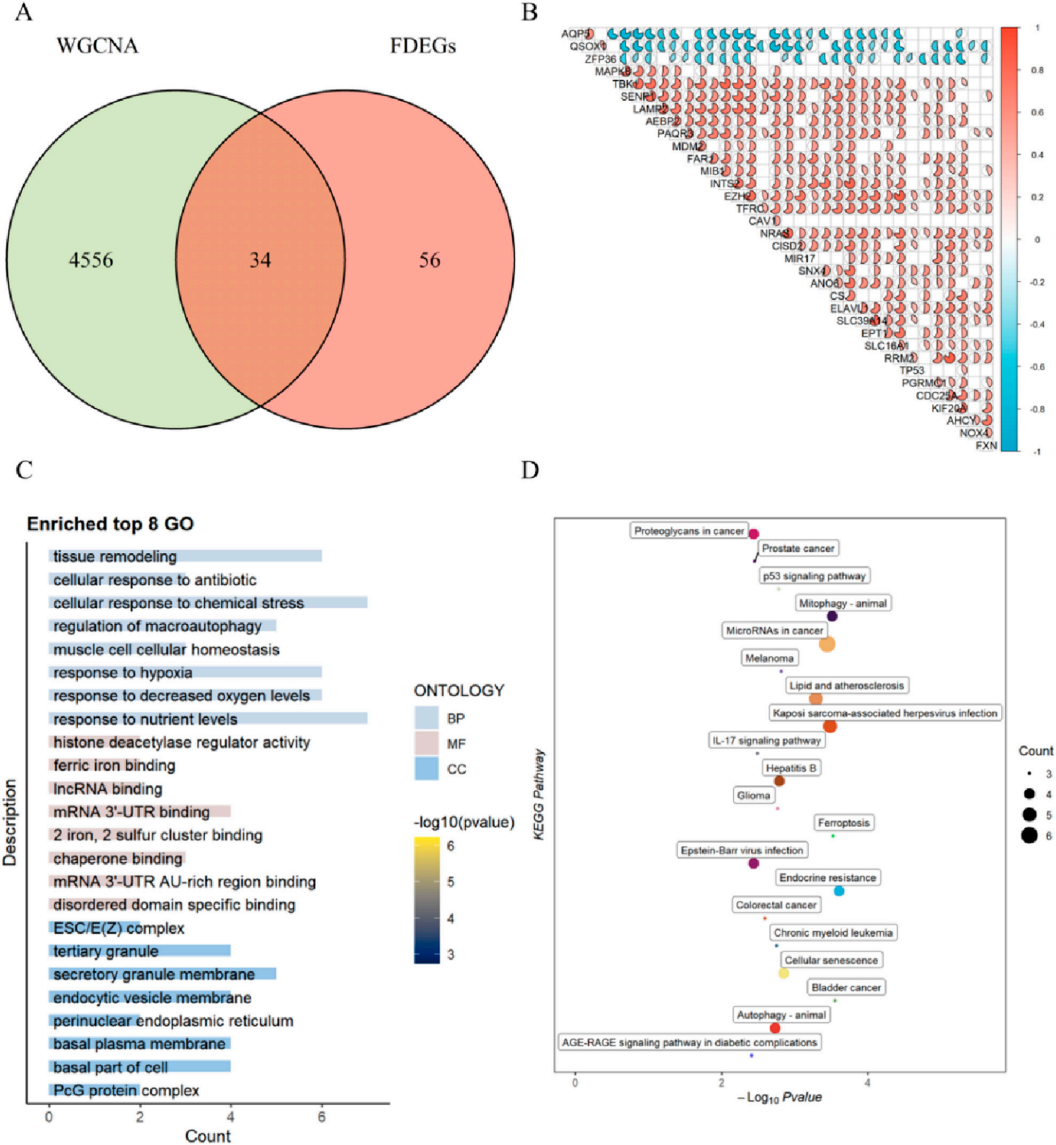
Correlation and Enrichment Analysis of DE-FRGs. **(A)** Venn diagram showing the intersection of differential ferroptosis-related genes and WGCNA module genes. **(B)** Correlation analysis of differential ferroptosis-related genes. **(C)** Bar plot of GO enrichment analysis for DE-FRGs. **(D)** Bubble plot of KEGG enrichment analysis for differential ferroptosis-related genes.

We performed the analysis on the standardized expression matrix of GSE12452 using the WGCNA package, which included 14,480 genes. A scale-free network was constructed by setting the soft threshold to 9 (R = 0.9) (Figure 3A). Dynamic tree cut was used to identify and merge similar gene modules, resulting in 9 gene modules (Figures 3B–D). By examining the Pearson correlation coefficients and p-values between each gene module and nasopharyngeal carcinoma, we found the brown module exhibited the strongest negative correlation with the tumor (r = −0.92), while the turquoise module showed the highest positive correlation with the tumor (r = 0.57) (Figure 3E). This study primarily focuses on module genes that are positively correlated with tumors. Therefore, based on the WGCNA analysis, we identified 4,590 nasopharyngeal carcinoma-associated genes from the turquoise module. We intersected these with 90 differentially expressed ferroptosis-related genes, identifying 34 differential ferroptosis-associated genes (Figure 4A).

### Correlation and functional enrichment analysis of differential ferroptosis-related genes

We performed a correlation analysis on the 34 genes obtained by intersecting the NPC-related module genes with DE-FRGs to explore the potential role of these ferroptosis-related differential genes in NPC progression. Some ferroptosis-related differential genes exhibited varying effects. For example, APQ5 and QSOX1 showed strong negative correlations, while NRAS and TBK1 displayed strong positive correlations (Figure 4B). Additionally, we conducted functional enrichment analysis on these ferroptosis-related differential genes. The results indicated that GO-BP was primarily associated with cellular response to chemical stress, response to nutrient levels, tissue remodeling, response to decreased oxygen levels and response to hypoxia. GO-MF revealed significant enrichment in mRNA 3′-UTR binding and chaperone binding. GO-CC showed that the genes were mainly associated with secretory granule membranes, endocytic vesicle membranes, basal plasma membrane, basal part of cell, and tertiary granules (Figure 4C). KEGG pathway analysis further demonstrated that these genes were significantly enriched in microRNAs in cancer, lipid and atherosclerosis, and Kaposi sarcoma-associated herpesvirus infection (Figure 4D).

### Identification of ferroptosis-related hub genes

**FIGURE 5:**
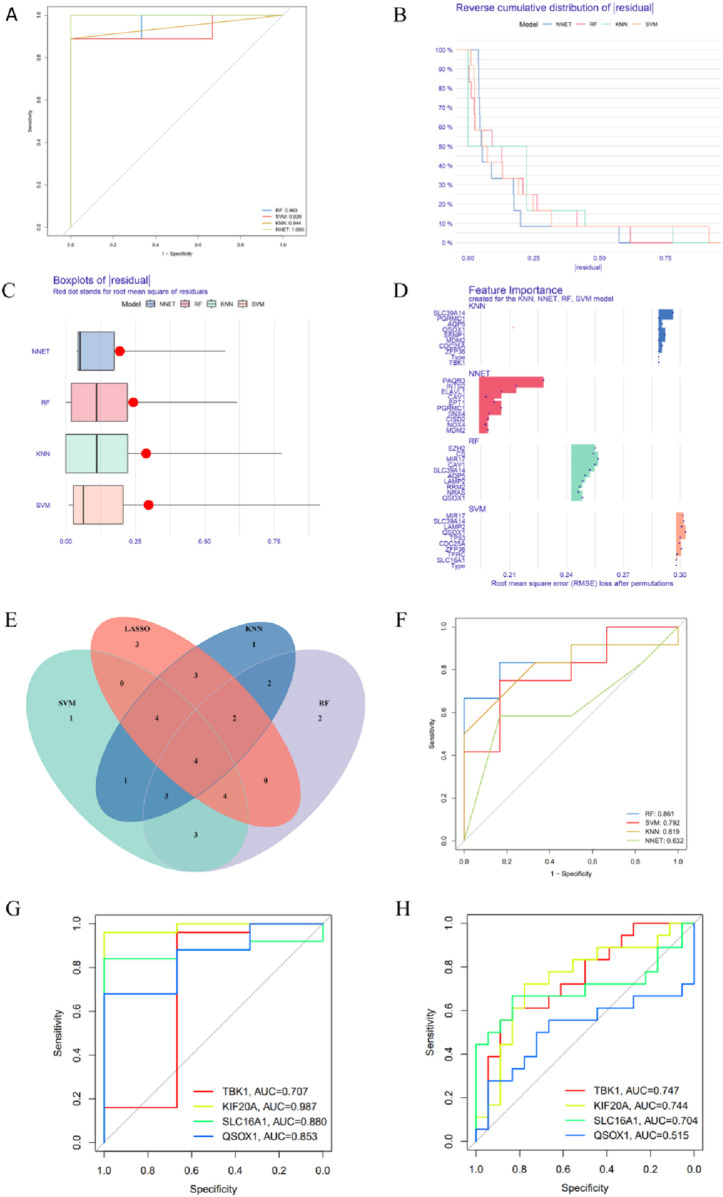
Identification and validation of hub genes. **(A)** ROC curves of four machine learning models. **(B)** Residual distribution plot of the machine learning models. **(C)** Boxplot of residual values from the machine learning models. **(D)** Histogram of feature contribution in the machine learning models. **(E)** Venn diagram of the four machine learning models. **(F)** ROC curve of the model validated on the combined dataset of GSE13597 and GSE53819. **(G)** ROC curves of hub genes in GSE13597. **(H)** ROC curves of hub genes in GSE53819.

We employed four distinct machine learning algorithms, including Random Forest (RF), Supported Vector Machine (SVM), Neural Networks (NNET), K-nearest Neighbor (KNN), to assist in identifying the core genes within the ferroptosis gene set. ROC curves were utilized to evaluate the performance of these four models (Figure 5A), all of which demonstrated high diagnostic efficiency. The AUC values for RF, SVM, KNN, and NNET were 0.963, 0.926, 0.944, and 1.000, respectively. Additionally, we assessed the stability of the models by plotting residual distribution and residual box plots (Figures 5B,C). These visualizations indicate that all four models are stable and hold practical value. The top 10 important feature genes for each model were ranked according to the root mean square error (RMSE) (Figure 5D). Furthermore, we extracted the top 20 significant feature genes from each model and performed an intersection, ultimately identifying four core genes: TBK1, KIF20A, SLC16A1, and QSOX1 (Figure 5E). Subsequently, we validated the diagnostic value of these four genes and the models using the GSE13597 and GSE53819 datasets (Figures 5F–H).

### Gene set enrichment analysis and gene set variation analysis

**FIGURE 6:**
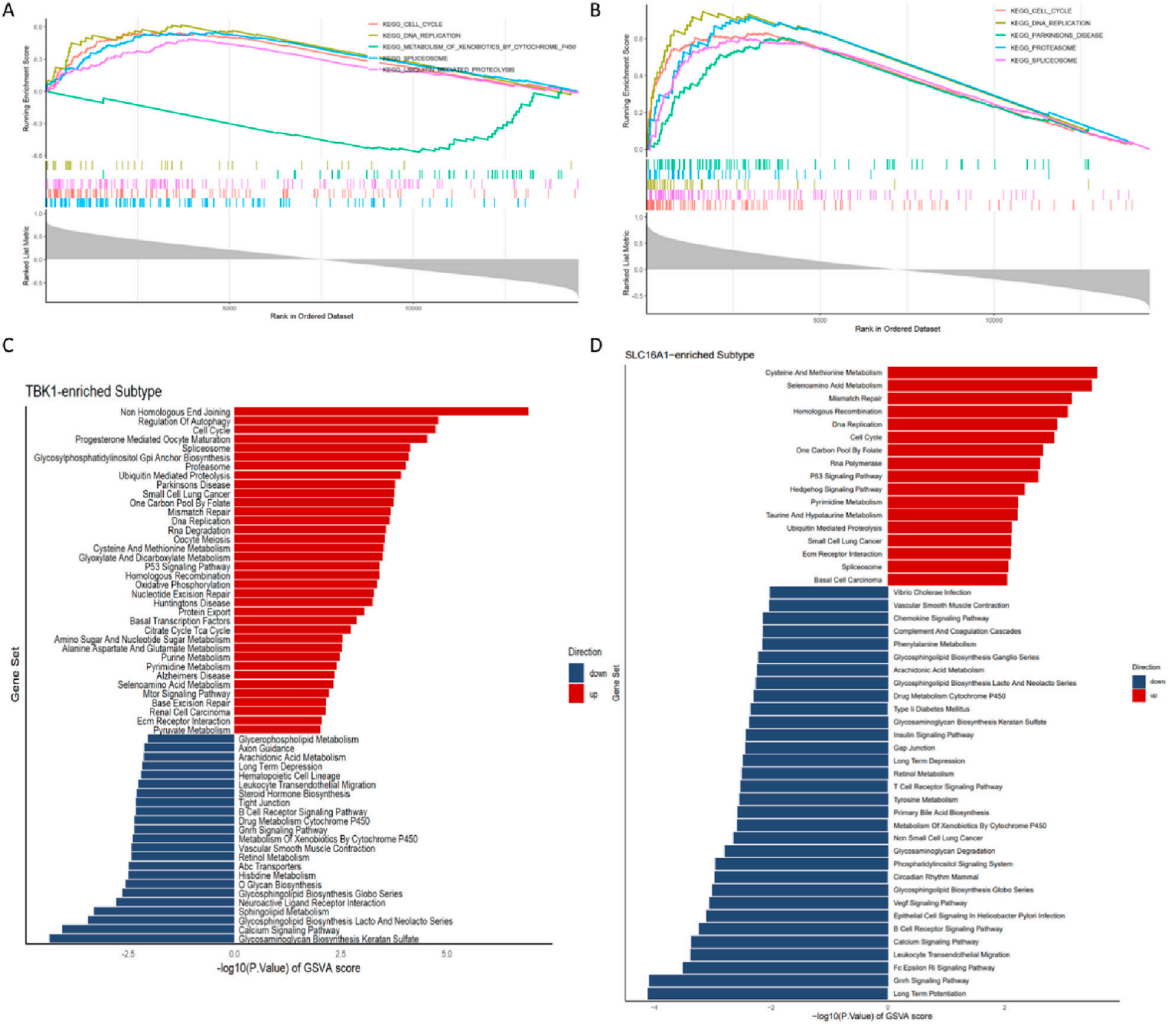
GSEA and GSVA of hub genes. **(A-B)** GSEA results of TBK1 and SLC16A1 genes. **(C-D)** GSVA results of TBK1 and SLC16A1 genes.

We conducted GSEA and GSVA and found that ubiquitin mediated proteolysis and non homologous end joining, cell cycle, DNA replication were activated in the TBK1 high-expression group, while metabolism of xenobiotics by cytochrome p450, Glycosaminoglycan Biosynthesis Keratan Sulfate were activated in the TBK1 low-expression group (Figures 6A,C). In the KIF20A high-expression group, pathways such as cell cycle, parkinsons disease and other pathways were activated. In the KIF20A low-expression group, no significant differences were observed in GSEA, whereas in GSVA, phosphatidylinositol signaling system and B cell receptor signaling pathway were activated (Supplementary Figure S2A,C). Additionally, cysteine, methionine metabolism, selenoamino acid metabolism, Pyrimidine metabolism, DNA replication and ribosome were activated in the SLC16A1 high-expression group, while long term potentiation and Gnrh signaling pathway were activated in the SLC16A1 low-expression group (Figures 6B,D). In the QSOX1 upregulation group, pathways such as glycosaminoglycan biosynthesis keratan sulfate and calcium signaling pathway were activated. In the QSOX1 downregulation group, pathways such as cell cycle and proteasome were activated (Supplementary Figure S2B,D).

### Immune infiltration analysis

**FIGURE 7:**
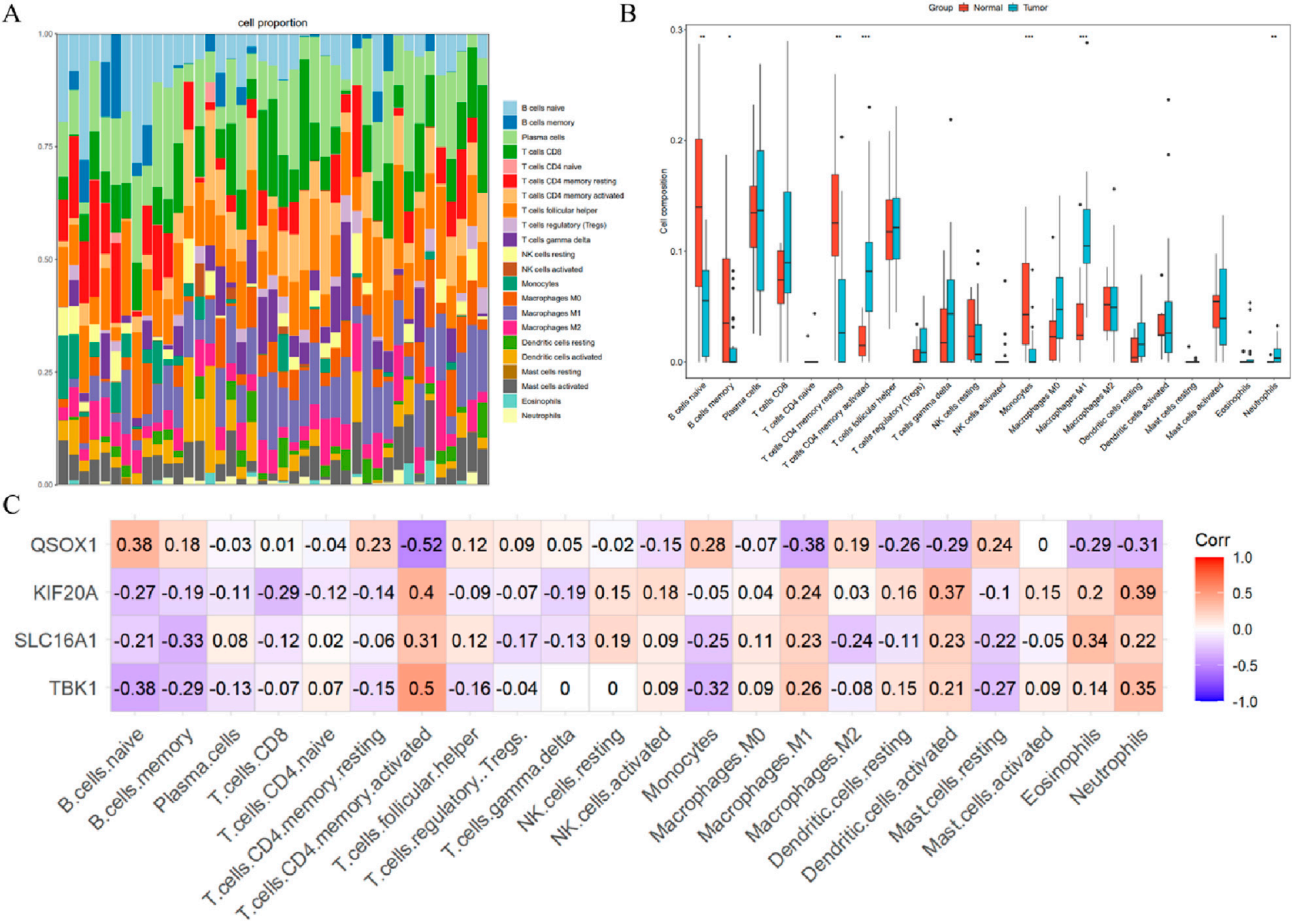
Immune Infiltration Analysis of GSE12452. **(A)** Proportions of 22 immune cell types in GSE12452 samples. **(B)** Differential levels of immune cell infiltration among the 22 immune cell types **(C)** Correlation analysis between hub genes and the 22 immune cell types.

To further investigate the correlation between the four hub genes and immune cells. In this study, the CIBERSORT algorithm was employed to infer immune cell characteristics and explore the relationship between immune regulation in NPC and diagnostic biomarkers with immune cell infiltration. Figure 7A illustrates the proportion of 22 immune cell types in each sample. Compared to the normal group, the NPC group exhibited higher proportions of CD4^+^ memory-activated T cells, M1 macrophages, and neutrophils, whereas the proportions of naive B cells, memory B cells, CD4^+^ memory resting T cells, and monocytes were lower (Figure 7B). Further analysis of the correlation between the expression of the four hub genes and the proportion of differentially infiltrated immune cell types revealed that TBK1, KIF20A, and SLC16A1 were strongly associated with CD4^+^ memory-activated T cells, M1 macrophages, and neutrophils, while QSOX1 showed a higher correlation with naive B cells, CD4^+^ memory resting T cells, and monocytes (Figure 7C). These findings suggest that ferroptosis-related genes may contribute to the pathological process of NPC through immune regulation.

### Single-cell analysis

**FIGURE 8:**
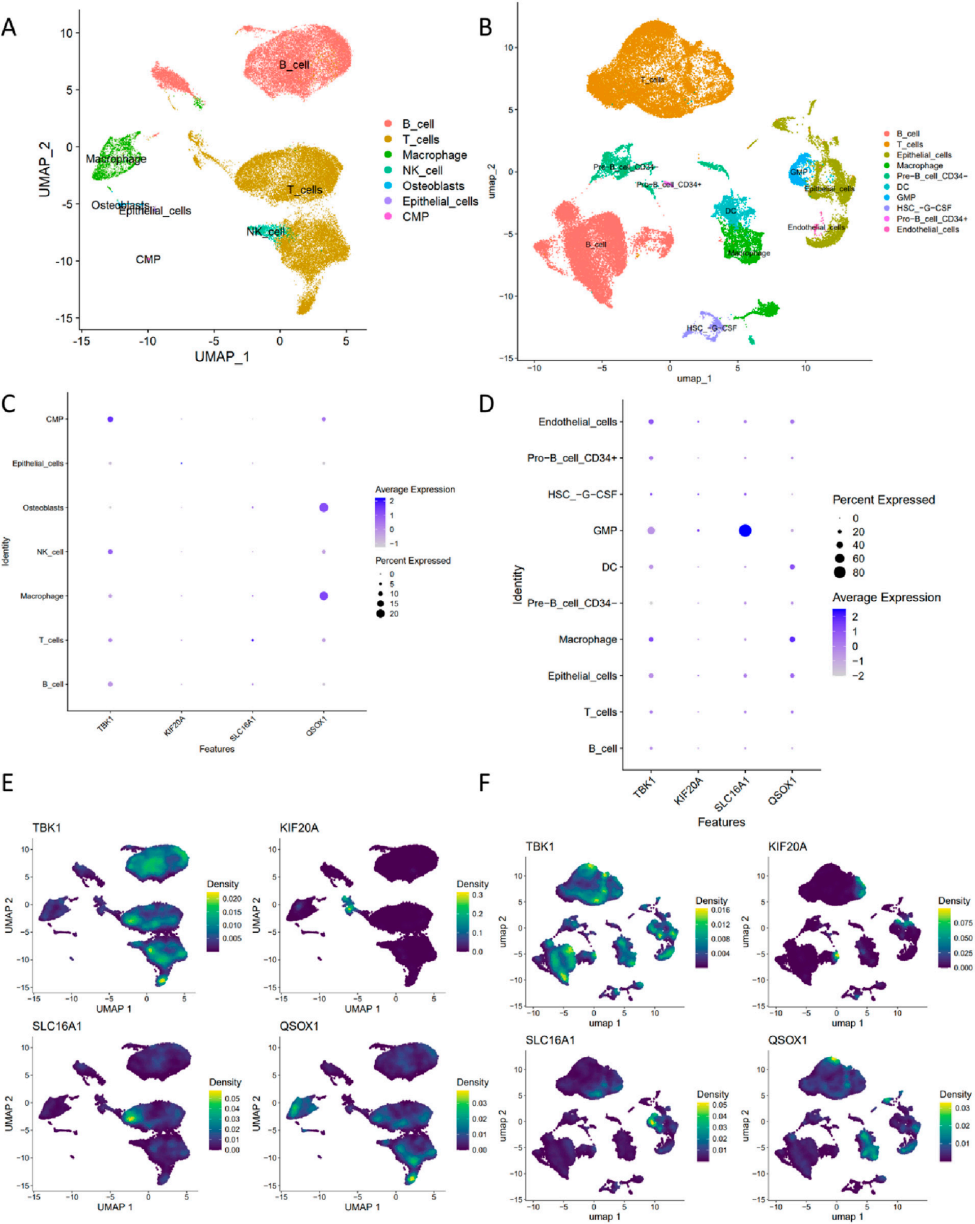
Single-cell analysis identifies the expression patterns of four ferroptosis hub genes. **(A)** UMAP plot showing seven cell types in GSE150825. **(B)** UMAP plot showing ten cell types in GSE150430. **(C)** Bubble plot displaying the expression levels of the four hub genes across different cell types in GSE150825. **(D)** Bubble plot displaying the expression levels of the four hub genes across different cell types in GSE150430. **(E)** Density distribution plot of the four hub genes in GSE150825. **(F)** Density distribution plot of the four hub genes in GSE150430.

To further investigate the expression patterns of four ferroptosis-related genes in cells and validate their associations with immune cells, we performed single-cell analysis (Figure 8). The results demonstrated that TBK1 and QSOX1 were widely distributed across the entire cell population, with expression observed in CMP, GMP, Pro_B_ cell_CD34+, DC, Macrophages, NK_cell, T cells, and B_cell. In contrast, KIF20A and SLC16A1 exhibited more restricted expression patterns. KIF20A showed modest expression in Epithelial_cells, GMP, Macrophages, and T_cells, while SLC16A1 was highly expressed in GMP and weakly expressed in epithelial cells, Osteoblasts, Endothelial cells, Macrophages, T_cells, and B_cells (Figures 8C–F).

### Drug prediction

Based on the DGIdb database, we identified potential targeted drugs associated with the four hub genes and analyzed the relationships between these drugs and the genes. Through database retrieval, we selected eight drugs related to TBK1, including Chembl:chembl 197335, NVP-TEA684, Entrectinib, Adavosertib, PF-56227, Cenisertib, CYC-116, and Tamatinib. For the SLC16A1 gene, three related drugs were identified: Sodium butyrate, Tetradecanoylphorbol acetate, and Butyric acid. No related drugs were found for the other two genes.

## Materials and methods

### Dataset extraction and pre-processing

The data used in this study were obtained from the Gene Expression Omnibus (GEO; https://www.ncbi.nlm.nih.gov/geo/) database. The GEO database includes three NPC-related microarray datasets (GSE12452, GSE13597, GSE53819) and one NPC-related single-cell sequencing dataset (GSE150825, GSE150430). Specifically, GSE12452 contains 31 NPC samples and 10 normal healthy nasopharyngeal tissue samples. GSE13597 includes 25 NPC patient samples and 3 samples from patients with no evidence of malignancy. GSE53819 includes 18 primary NPC tumor samples and 18 non-cancerous nasopharyngeal tissue samples. GSE150825 contains single-cell sequencing data from 14 NPC patients and GSE150430 Contains 15 primary nasopharyngeal carcinoma tumor samples and one normal sample. The raw data were processed using R (version 4.2.2). Initially, the microarray data were processed to remove batch effects, resulting in an expression matrix. Ferroptosis-related genes, including driver genes, inhibitor genes, and marker genes, were downloaded from the FerrDb database (www.zhounan.org/ferrdb/), totaling 484 ferroptosis-related genes. This study adhered to the usage guidelines of both the GEO and FerrDb databases.

### Screening of ferroptosis-related differential genes

The limma package in R was used to screen for differentially expressed genes in GSE12452, with the selection criteria set as |Log2 fold change| > 0.5 and an adjusted P-value <0.05 for differential genes (Li et al., 2022). The results of the differential analysis were visualized using ggplot2. Subsequently, the intersection of these differential genes with ferroptosis-related genes was identified, yielding the ferroptosis-related differential genes, which were visualized using a Venn diagram.

### Functional enrichment analysis

The clusterProfiler package in R was used to perform GO and KEGG enrichment analysis on the selected DE-FRGs (Yu et al., 2012). A P-value <0.05 was considered statistically significant for differential enrichment.

### Weighted gene co-expression network analysis

The WGCNA package (Langfelder and Horvath, 2008) was used to perform sample clustering on the normalized microarray expression matrix, followed by the removal of outlier samples. After constructing a scale-free network based on the optimal soft threshold, gene clustering analysis was performed to form gene modules, represented by different colors. Gene modules were identified using dynamic tree cut methods, and modules highly positively correlated with NPC were selected. The intersection of the selected module genes and ferroptosis-related differential genes was then analyzed to obtain DE-FRGs which were strongly associated with disease characteristics.

### Construction and validation of diagnostic models based on four machine learning algorithms

This study utilized four machine learning models: support vector machine model (SVM), random forest model (RF), k-nearest neighbors (KNN), and neural network (NNET). The DALEX package in R was used to interpret the residual distribution and feature importance of the machine learning models. To assess the reliability of the disease diagnostic models, ROC curves were constructed using the pROC package, and the intersection of genes identified by the four machine learning algorithms was visualized using the VennDiagram package, which revealed four hub genes. Finally, the diagnostic performance of these four hub genes was validated using ROC curves in an independent validation cohort.

### GSEA and GSVA of hub genes

To more accurately investigate the pathway activation differences caused by the differential expression of hub genes in diseases, this study divided the samples into two groups based on the median expression level of the hub genes: high expression and low expression. GSEA and GSVA enrichment analyses were performed using pathway datasets from the MSigDB database (Kuang et al., 2023) (https://www.gsea-msigdb.org/gsea/msigdb). Both the GSEA and GSVA algorithms are based on gene expression levels and calculate the differences in pathway activation between the two groups.

### Construction of gene-drug regulatory network

The Drug-Gene Interaction database (DGIdb) is a free database that provides information on the interactions between genes and known or potential drugs. The four key ferroptosis-related genes identified in this study were input into DGIdb to obtain drug information related to their interactions. This information was then used to construct a gene-drug regulatory network.

### Immune infiltration analysis

The CIBERSORT algorithm was used to analyze the infiltration proportions of 22 common immune cell types in different samples (Newman et al., 2019). Differential analysis of immune infiltration between the normal and disease groups was performed, and violin plots were generated using the vioplot package, with a threshold of P < 0.05. The correlation between key ferroptosis-related genes and the 22 immune cell types was analyzed using the limma, reshape2, tidyverse, and ggplot2 packages, and a correlation heatmap was constructed.

### Single-cell analysis

We integrated transcriptomic data from the GSE150825 dataset. Single-cell analysis was conducted using the R packages including Seurat, tidyverse, Nebulosa, and Matrix.

### Statistical methods

Categorical variables were analyzed using either the Chi-square test or Fisher’s exact test, as appropriate. The diagnostic accuracy of the four hub genes was evaluated by receiver operating characteristic (ROC) curve analysis, with results expressed as the area under the ROC curve (AUROC) and 95% confidence intervals (CIs). The associations between hub genes and immune cells or immune checkpoint genes were examined using Spearman’s rank correlation or Pearson correlation coefficient analysis. All statistical analyses were performed using R software (version 4.2.2). A two-tailed P-value less than 0.05 was considered statistically significant.

## Discussion

Ferroptosis, as a unique form of cell death, plays a critical role in the development of NPC. Inducing ferroptosis not only suppresses tumor growth but also has the potential to enhance the response to immunotherapy and overcome resistance to existing cancer treatments (Lei et al., 2024). However, the exact role and specific molecular mechanisms of ferroptosis in NPC remain unclear and require further investigation.

In this study, we performed bioinformatics analysis by combining NPC microarray data with ferroptosis-related genes. We identified 34 ferroptosis-related differential genes associated with NPC. Enrichment analysis revealed that these genes are predominantly involved in pathways related to the response to hypoxia, the response to reduced oxygen levels, the response to chemical stress, and lipid metabolism and atherosclerosis pathways. Subsequently, four machine learning methods were employed to screen for hub genes associated with ferroptosis, resulting in the identification of four ferroptosis-related hub genes: TBK1, KIF20A, SLC16A1, and QSOX1. Further exploration of the relationships between these hub genes and immune cells was conducted, followed by drug prediction to investigate their interactions with drugs. NPC is known to be associated with immune system dysfunction (Chen et al., 2018). The CIBERSORT method was used to evaluate changes in immune infiltration, revealing that TBK1, KIF20A, and SLC16A1 were strongly correlated with CD4 memory-activated T cells, M1 macrophages, and neutrophils. CD4 memory T cells possess an activated phenotype, enhanced proliferation potential, and rapid cytokine secretion capability (Stockinger et al., 2004). M1 macrophages are capable of promoting inflammatory responses by producing pro-inflammatory factors such as IL-6, IL-12, and tumor necrosis factor (TNF) (Murray et al., 2014). M1 macrophages are typically considered tumor-killing macrophages, primarily contributing to anti-tumor immunity and immune enhancement (Yunna et al., 2020). Neutrophils play roles in inducing DNA damage, promoting angiogenesis, immune suppression, and inhibiting cancer growth (Xiong et al., 2021). Therefore, through the involvement of immune cells, we can gain a more intuitive understanding of the role of the hub genes we identified in the tumor context.

Immunoinfiltration analysis revealed that the expression of TBK1, KIF20A, and SLC16A1 was positively correlated with CD4^+^ memory-activated T cells, M1 macrophages, and neutrophils. Further single-cell analysis confirmed that TBK1 and QSOX1 were predominantly expressed in CMP, GMP, Pro_B_ cell_CD34+, DC, macrophages, NK_cell, T_cells, and B cells, while SLC16A1 was mainly expressed in GMP. TBK1 has been shown to mediate the ORF5/IRF4 axis during M-CSF-induced macrophage polarization (Li et al., 2024). Elevated TBK1 levels may promote proinflammatory signaling, linking TBK1 to inflammatory macrophages. SLC161, by facilitating immunosuppressive cell infiltration, acts as a bridge between tumor metabolism and immune evasion (Zhu et al., 2022), which may further explain its pro-tumorigenic role. Although experimental validation is required, these findings suggest that ferroptosis-related genes may shape the immune landscape of nasopharyngeal carcinoma (NPC) through metabolic and immunoregulatory pathways. This provides valuable insights for future research and could aid clinicians in developing precision treatment strategies for patients.

Studies have shown that TBK1 inhibition not only suppresses cancer progression by directly inhibiting cancer cell proliferation and survival but also suppresses cancer development by activating anti-tumor T cell immunity (Runde et al., 2022). TBK1 is a critical node in the innate immune pathway and mediates anti-tumor immunity by activating innate immune responses. TBK1 is involved in various aspects of tumorigenesis, including supporting tumor angiogenesis, mediating tumor-associated autophagy, regulating the cell cycle and mitosis, and inducing epithelial-mesenchymal transition (EMT) (WanG et al., 2024). In both GSEA and GSVA analyses, pathways such as cell cycle, DNA replication, metabolism of xenobiotics by cytochrome p450, ubiquitin mediated proteolysis and non homologous end joining were activated in TBK1, indicating that TBK1 plays an important role in NPC by mediating NPC-associated autophagy, regulating cell cycle and mitosis, and inducing epithelial-mesenchymal transition. KIF20A suppresses cancer cell proliferation, migration, and invasion. As a member of the kinesin family, KIF20A contributes to cancer progression by regulating cell division (Jin et al., 2023). In GSEA analysis, we found that the cell cycle pathway was activated in the high-expression group of KIF20A, further validating the role of KIF20A in NPC progression through its involvement in cell division. SLC16A1 plays an important role in cancer metabolism, promoting cancer progression and metastasis (Zhang et al., 2021). Through GSEA and GSVA analyses, we observed activation of pathways such as cysteine, methionine metabolism, selenoamino acid metabolism, pyrimidine metabolism, DNA replication and ribosome in the high-expression group of SLC16A1. These findings indicate that SLC16A1 contributes to NPC metabolism and metastasis by influencing DNA replication, ribosome, and metabolic pathways like cysteine and methionine metabolism, selenoamino acid metabolism, and pyrimidine metabolism. QSOX1 expression is associated with tumor cell invasion, tumor grading, and abnormal extracellular matrix protein deposition (Millar-Haskell et al., 2022). The QSOX1 gene plays a role in the progression of NPC by glycosaminoglycan biosynthesis keratan sulfate and calcium signaling pathway in NPC.

Accumulating evidence indicates that these four hub genes play critical roles in various cancers. Chen et al. demonstrated that TBK1 regulates malignant behaviors of bladder cancer cells via Akt signaling, providing novel insights into potential therapeutic targets for this disease (Chen et al., 2017). Yamashita et al. identified KIF20A as a melanoma-associated antigen with diagnostic and prognostic potential (Yamashita et al., 2012). Zou et al. further established KIF20A as both a prognostic factor and therapeutic target for endocrine therapy-resistant breast cancer (Zou et al., 2014). Huang et al. revealed the oncogenic role of SLC16A1 in cholangiocarcinoma, highlighting its therapeutic relevance (Huang et al., 2024). Zhang et al. reported that SLC16A1 overexpression serves as a biomarker for poor prognosis in urinary system cancers (Zhang et al., 2021). Baek et al. associated elevated QSOX1 expression with tumor aggressiveness and high-grade prostate cancer, suggesting its utility as a biomarker and therapeutic target (Baek et al., 2018). Pernodet et al. proposed QSOX1 as a favorable prognostic biomarker in breast cancer and demonstrated its tumor-suppressive effects in mammary carcinogenesis (Pernodet et al., 2012). In summary, future studies should validate our findings in non-NPC models and different populations to assess broader applicability.

Through drug prediction base on the four hub genes, several potential therapeutic agents were identified as follows: Chembl:chembl 197335, NVP-TEA684, Entrectinib, Adavosertib, PF-56227, Cenisertib, CYC-116, and Tamatinib. Entrectinib (Rozlytrek^®^) is an oral selective inhibitor of the tyrosine kinases tropomyosin receptor kinases (Trk)A/B/C [encoded by the genes neurotrophic tyrosine receptor kinase (NTRK) 1, 2 and 3, respectively], c-ros oncogene 1 (ROS1) and anaplastic lymphoma kinase (ALK) with central nervous system (CNS) activity developed by Roche for the treatment of various solid tumours harbouring NTRK1/2/3 or ROS1 gene fusions (Al-Salama and Keam, 2019). The other drugs are still unapproved and will require further validation through clinical trials.

In summary, we employed bioinformatics approaches to identify four hub genes related to ferroptosis and explored their roles in disease development through a literature review. However, due to the lack of critical clinical characteristics associated with patients, we were unable to perform survival analysis or further investigate the relationship between these four hub genes and prognosis, which limits their utility in assisting clinicians with NPC prognosis prediction. We recommend that subsequent studies incorporate clinical data from nasopharyngeal carcinoma patients to facilitate more comprehensive analyses, thereby enabling more accurate prognostic prediction and the development of precisely personalized treatment strategies for clinical practice. Overall, our research enhances the understanding of the molecular mechanisms of ferroptosis in the development of NPC and provides valuable insights to help clinicians develop personalized treatment strategies for patients.

## Data Availability

The original contributions presented in the study are included in the article/Supplementary Material, further inquiries can be directed to the corresponding authors.
