# Supplementary material for: Identification of key ferroptosis-related genes and therapeutic target in nasopharyngeal carcinoma
Source: Front Genet. 2025 Jul 31;16:1595456. doi: 10.3389/fgene.2025.1595456 (PMC12350130; doi:10.3389/fgene.2025.1595456)
Supplement: Supplementary file 1 [file DataSheet2.docx]

1. **This is a comparison of the AUC values of the 4 machine learning algorithms for the training and validation sets.**

**Supplementary table 1**

|  | GSE12452 | GSE13597 and GSE53819 |
| --- | --- | --- |
| RF | 0.963 | 0.861 |
| SVM | 0.926 | 0.792 |
| KNN | 0.944 | 0.819 |
| NNET | 1.000 | 0.632 |

1. **This is a table of the results of the DGIdb analysis of TBK1.**

**Supplementary table 2**

| gene | drug | regulatory approval | indication | interaction score |
| --- | --- | --- | --- | --- |
| TBK1 | CHEMBL:CHEMBL1997335 | Not Approved |  | 0.1562728310586145 |
| TBK1 | NVP-TAE684 | Not Approved |  | 0.06372290198506614 |
| TBK1 | ENTRECTINIB | Approved |  | 0.1151484018326633 |
| TBK1 | ADAVOSERTIB | Not Approved | antineoplastic agent | 0.2344092465879218 |
| TBK1 | PF-562271 | Not Approved |  | 0.06077276763390567 |
| TBK1 | CENISERTIB | Not Approved | antineoplastic agent | 0.05562253308865942 |
| TBK1 | CYC-116 | Not Approved |  | 0.06766452478826611 |
| TBK1 | TAMATINIB | Not Approved |  | 0.08103035684520755 |

1. **This is a table of the results of the DGIdb analysis of SLC16A1.**

**Supplementary table 3**

| gene | drug | regulatory approval | indication |
| --- | --- | --- | --- |
| SLC16A1 | SODIUM BUTYRATE | Not Approved |  |
| SLC16A1 | TETRADECANOYLPHORBOL ACETATE | Not Approved |  |
| SLC16A1 | BUTYRIC ACID | Not Approved |  |

1. **This is a bar graph of the GO enrichment analysis of the 3405 differential genes in the GSE12452 dataset.**


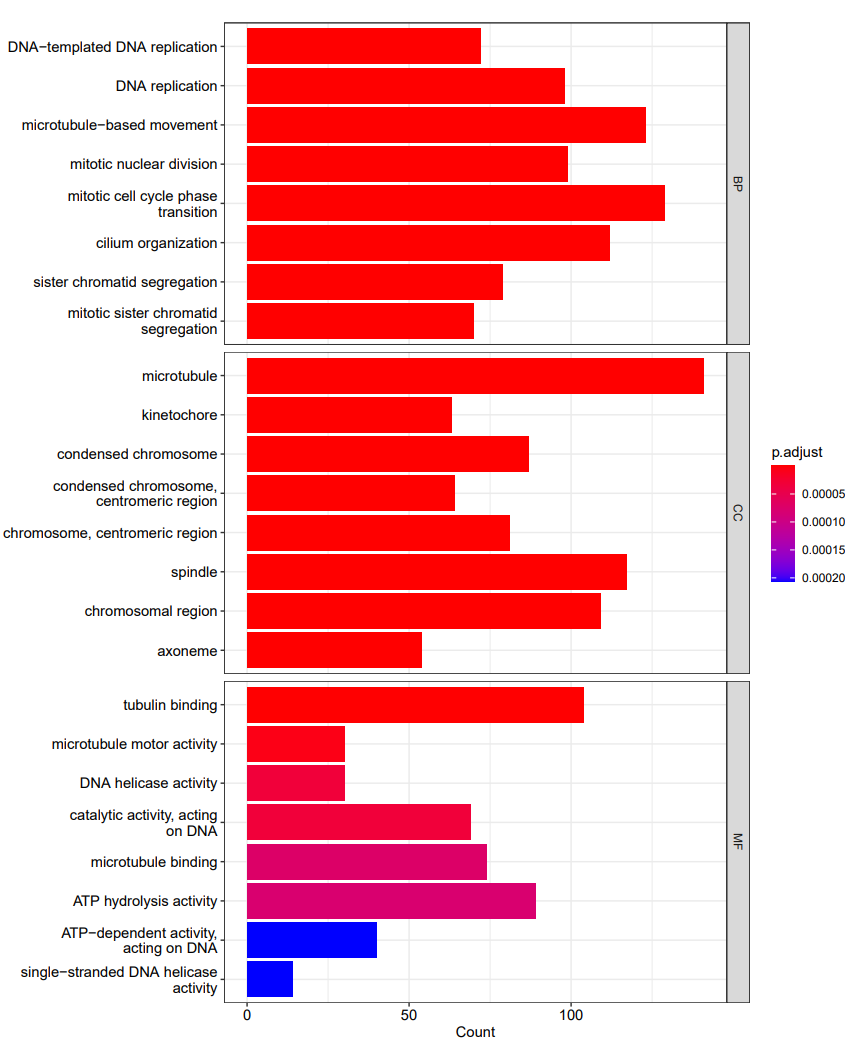


Supplementary Figure 1. Bar graph of GO enrichment analysis of GSE12452 differential gene

1. **This is the GSEA and GSVA analysis chart for KIF20A and QSOX1.**

**
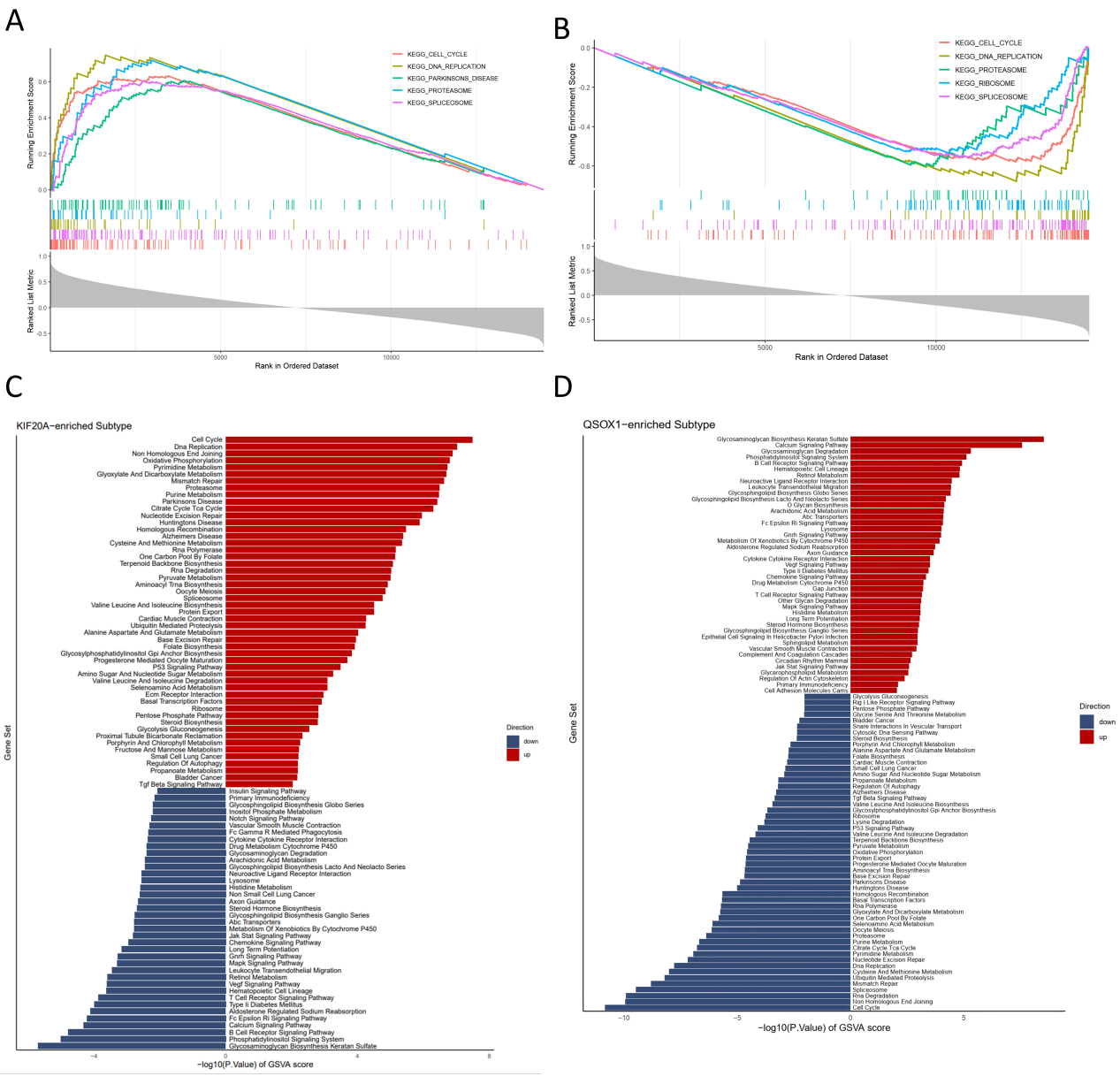
**

Supplementary Figure 2. GSEA and GSVA of hub genes. The above is the GSEA analysis of the hub genes KIF20A and QSOX1, and the below is the GSVA analysis of the hub genes KIF20A and QSOX1.
